# A triclinic polymorph of hexa­nedioic acid

**DOI:** 10.1107/S1600536809006448

**Published:** 2009-02-28

**Authors:** Hoong-Kun Fun, Suchada Chantrapromma

**Affiliations:** a X-ray Crystallography Unit, School of Physics, Universiti Sains Malaysia, 11800 USM, Penang, Malaysia; b Crystal Materials Research Unit, Department of Chemistry, Faculty of Science, Prince of Songkla University, Hat-Yai, Songkhla 90112, Thailand

## Abstract

Hexane­dioic acid (or adipic acid), C_6_H_10_O_4_, crystallizes with two crystallographically independent half-mol­ecules in the asymmetric unit of the triclinic unit cell, space group *P* 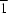, as each mol­ecule lies across a crystallographic inversion centre. A monoclinic polymorph has been reported previously, most recently by Ranganathan, Kulkarni & Rao [*J. Phys. Chem. A*, (2003), **107**, 6073–6081]. The mol­ecules adopt the expected zigzag structure and are linked *via* centrosymmetric pairs of O—H⋯O hydrogen bonds, forming infinite one-dimensional chains along [011]. These chains are stacked along the *a* axis. The crystal is further stabilized by weak C—H⋯O inter­actions.

## Related literature

For bond-length data, see Allen *et al.* (1987 ▶). For related structures, see, for example: Ranganathan *et al.* (2003 ▶); Srinivasa Gopalan *et al.* (1999 ▶, 2000 ▶). For general background to the influence of hydrogen bonding on phase transitions, see, for example: Chantrapromma *et al.* (2006 ▶); Dunitz (1991 ▶); Fun *et al.* (2003 ▶, 2006 ▶); How *et al.* (2005 ▶). For the stability of the temperature controller used in the data collection, see: Cosier & Glazer (1986 ▶).
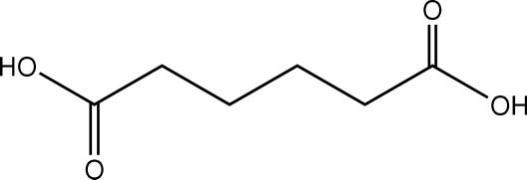


## Experimental

### 

#### Crystal data

C_6_H_10_O_4_
*M* *_r_* = 146.14
Triclinic, 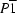

*a* = 6.7666 (5) Å
*b* = 6.9992 (5) Å
*c* = 7.7180 (5) Å
α = 93.794 (4)°
β = 104.321 (4)°
γ = 102.689 (4)°
*V* = 342.70 (4) Å^3^
*Z* = 2
Mo *K*α radiation
μ = 0.12 mm^−1^
*T* = 100 K
0.55 × 0.11 × 0.06 mm

#### Data collection

Bruker APEXII CCD area-detector diffractometer
Absorption correction: multi-scan (*SADABS*; Bruker, 2005 ▶) *T*_min_ = 0.847, *T*_max_ = 0.993
7773 measured reflections
1553 independent reflections
1419 reflections with *I* > 2σ(*I*)
*R*_int_ = 0.027

#### Refinement

*R*[*F* ^2^ > 2σ(*F* ^2^)] = 0.037
*wR*(*F* ^2^) = 0.094
*S* = 1.09
1553 reflections
91 parameters
H-atom parameters constrained
Δρ_max_ = 0.32 e Å^−3^
Δρ_min_ = −0.20 e Å^−3^

### 

Data collection: *APEX2* (Bruker, 2005 ▶); cell refinement: *APEX2*; data reduction: *SAINT* (Bruker, 2005 ▶); program(s) used to solve structure: *SHELXTL* (Sheldrick, 2008 ▶); program(s) used to refine structure: *SHELXTL*; molecular graphics: *SHELXTL*; software used to prepare material for publication: *SHELXTL* and *PLATON* (Spek, 2009 ▶).

## Supplementary Material

Crystal structure: contains datablocks global, I. DOI: 10.1107/S1600536809006448/sj2583sup1.cif

Structure factors: contains datablocks I. DOI: 10.1107/S1600536809006448/sj2583Isup2.hkl

Additional supplementary materials:  crystallographic information; 3D view; checkCIF report

## Figures and Tables

**Table 1:** Hydrogen-bond geometry (Å, °)

*D*—H⋯*A*	*D*—H	H⋯*A*	*D*⋯*A*	*D*—H⋯*A*
O2*A*—H2*OA*⋯O1*A*^i^	0.82	1.82	2.6397 (13)	172
O2*B*—H2*OB*⋯O1*B*^ii^	0.82	1.82	2.6421 (13)	174
C2*A*—H2*AB*⋯O2*A*^iii^	0.97	2.58	3.5415 (16)	171

Symmetry codes: (i) 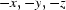; (ii) 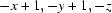; (iii) 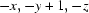.

**Fig. 1.**
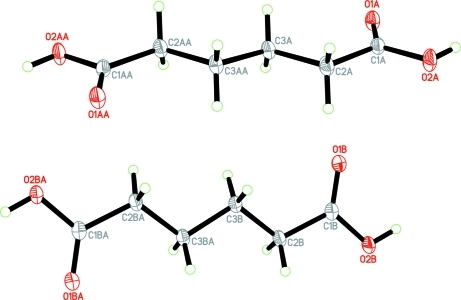
The molecular structure of the title compound, showing 50% probability displacement ellipsoids and the atomic numbering. [Symmetry code: (AA) -x, -y+1, -z+1 and (BA) -x+1, -y+2, -z+1].

**Fig. 2.**
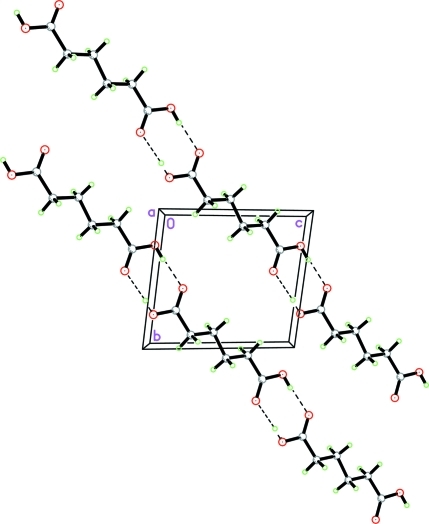
The crystal packing of the title compound, viewed down the a axis showing one-dimensional chains along the [0 1 1] direction. Hydrogen bonds are shown as dashed lines.

## supplementary crystallographic information

### Comment

Structural investigation of crystalline solids undergoing phase transition has been an interesting area of research. Molecular solids are more interesting in that they often crystallize in different structural forms and exhibit polymorphic transformations (Dunitz, 1991). We have previously reported reversible phase transitions due to hydrogen bonds in some organic compounds (Chantrapromma *et al.*, 2006; Fun *et al.*, 2003; 2006); How *et al.*, 2005). Aliphatic dicarboxylic acids form an interesting class of organic compounds for hydrogen bonding and phase transition studies. In the course of our research on the influence of hydrogen bonding on phase transitions, we have found that adipic acid exists in both monoclinic and triclinic polymorphs. The triclinic form does not undergo a phase transition in sharp contrast to the behaviour of the monoclinic form (Srinivasa Gopalan *et al.*, (1999). We report herein the crystal structure of the triclinic polymorph of adipic acid (I).

The crystal structure of the hexanedioic acid or adipic acid was previously reported by Ranganathan *et al.*, (2003) and Srinivasa Gopalan *et al.*, (1999, 2000) in the monoclinic space group *P*21/c. It was found that adipic acid exhibits a phase transition at around 136 K (Srinivasa Gopalan *et al.*, 1999) and does not exhibit polymorphism (Srinivasa Gopalan *et al.*, 2000). However, in the present work, we have found that adipic acid actually does exhibit polymorphisim in which the compound crystallized out in the triclinic space group *P* -1.

In the structure of (I), Fig. 1, each of the two unique adipic acid molecules, C_6_H_10_O_4_, lies across a different crystallographic inversion centre. There are two crystallographically independent half molecules in the asymmetric unit, *A* and *B*, with slightly different bond lengths and bond angles. The molecules exist in an zigzag form. Atoms O1A, O2A, C1A, C2A and C3 lie on the same plane in one molecule with a maximum deviation of 0.006 (1) Å for C1A while atoms O1B, O2B, C1B and C2B in the other molecule are also coplanar with a maximum deviation -0.005 (1) Å for atom C1B. The interplanar angle between these two planes is 61.14 (7)°. Bond lengths and angles in the title compound are within normal ranges (Allen *et al.*, 1987) and comparable to those in related structures (Ranganathan *et al.*, 2003; Srinivasa Gopalan *et al.*, 1999; 2000).

In the crystal packing (Fig. 2), the molecules are linked by centrosymmetric pairs of  O—H···O hydrogen bonds forming infinite one-dimensional chains along the [0 1 1] directions and these molecular chains are stacked along the *a* axis. The crystal is stablized by O—H···O hydrogen bonds and weak C—H···O interactions (Table 1). It is interesting to note that this triclinic polymorph has fewer O—H···O hydrogen bonds and weak C—H···O interactions in comparison to the monoclinic form (Srinivasa Gopalan *et al.*, 1999; 2000).

### Experimental

Adipic acid was obtained commercially (Fluka, Germany). Single crystals of adipic acid were grown by slow evaporation of ethyl acetate solution at room temperature.

### Refinement

All the H atoms were positioned geometrically and refined using a riding model with C—H = 0.93–0.96 Å and O—H = 0.82 Å. The *U*_iso_ values were constrained to be -1.2*U*_eq_ of the carrier atom for all hydrogen atoms. The highest residual electron density peak is located at 0.72 Å from C1A and the deepest hole is located at 0.70 Å from C3A.

### Crystal data

**Table:**

C_6_H_10_O_4_	*Z* = 2
*M**_r_* = 146.14	*F*(000) = 156
Triclinic, *P*1	*D*_x_ = 1.416 Mg m^−^^3^
Hall symbol: -P 1	Mo *K*α radiation, λ = 0.71073 Å
*a* = 6.7666 (5) Å	Cell parameters from 1553 reflections
*b* = 6.9992 (5) Å	θ = 2.8–27.5°
*c* = 7.7180 (5) Å	µ = 0.12 mm^−^^1^
α = 93.794 (4)°	*T* = 100 K
β = 104.321 (4)°	Needle, colorless
γ = 102.689 (4)°	0.55 × 0.11 × 0.06 mm
*V* = 342.70 (4) Å^3^	

### Data collection

**Table:**

Bruker APEXII CCD area-detector diffractometer	1553 independent reflections
Radiation source: sealed tube	1419 reflections with *I* > 2σ(*I*)
graphite	*R*_int_ = 0.027
φ and ω scans	θ_max_ = 27.5°, θ_min_ = 2.8°
Absorption correction: multi-scan (*SADABS*; Bruker, 2005)	*h* = −8→8
*T*_min_ = 0.847, *T*_max_ = 0.993	*k* = −9→9
7773 measured reflections	*l* = −10→10

### Refinement

**Table:**

Refinement on *F*^2^	Primary atom site location: structure-invariant direct methods
Least-squares matrix: full	Secondary atom site location: difference Fourier map
*R*[*F*^2^ > 2σ(*F*^2^)] = 0.037	Hydrogen site location: inferred from neighbouring sites
*wR*(*F*^2^) = 0.094	H-atom parameters constrained
*S* = 1.09	*w* = 1/[σ^2^(*F*_o_^2^) + (0.0402*P*)^2^ + 0.1411*P*] where *P* = (*F*_o_^2^ + 2*F*_c_^2^)/3
1553 reflections	(Δ/σ)_max_ = 0.001
91 parameters	Δρ_max_ = 0.32 e Å^−^^3^
0 restraints	Δρ_min_ = −0.20 e Å^−^^3^

### Special details

**Table:**

Experimental. The crystal was placed in the cold stream of an Oxford Cryosystems Cobra open-flow nitrogen cryostat (Cosier & Glazer, 1986) operating at 100.0 (1) K.
Geometry. All e.s.d.'s (except the e.s.d. in the dihedral angle between two l.s. planes) are estimated using the full covariance matrix. The cell e.s.d.'s are taken into account individually in the estimation of e.s.d.'s in distances, angles and torsion angles; correlations between e.s.d.'s in cell parameters are only used when they are defined by crystal symmetry. An approximate (isotropic) treatment of cell e.s.d.'s is used for estimating e.s.d.'s involving l.s. planes.
Refinement. Refinement of *F*^2^ against ALL reflections. The weighted *R*-factor *wR* and goodness of fit *S* are based on *F*^2^, conventional *R*-factors *R* are based on *F*, with *F* set to zero for negative *F*^2^. The threshold expression of *F*^2^ > σ(*F*^2^) is used only for calculating *R*-factors(gt) *etc*. and is not relevant to the choice of reflections for refinement. *R*-factors based on *F*^2^ are statistically about twice as large as those based on *F*, and *R*- factors based on ALL data will be even larger.

### Fractional atomic coordinates and isotropic or equivalent isotropic displacement parameters (Å^2^)

**Table:**

	*x*	*y*	*z*	*U*_iso_*/*U*_eq_	
O1A	0.07345 (14)	0.10624 (13)	0.21778 (12)	0.0182 (2)	
O2A	−0.11534 (14)	0.20271 (13)	−0.02751 (12)	0.0198 (2)	
H2OA	−0.0961	0.1042	−0.0779	0.030*	
C1A	−0.02548 (19)	0.22010 (17)	0.14771 (16)	0.0156 (3)	
C2A	−0.0623 (2)	0.39330 (18)	0.24950 (16)	0.0174 (3)	
H2AA	−0.2127	0.3787	0.2269	0.021*	
H2AB	−0.0057	0.5124	0.2026	0.021*	
C3A	0.03484 (19)	0.41932 (17)	0.45233 (16)	0.0167 (3)	
H3AA	0.1794	0.4448	0.4777	0.020*	
H3AB	−0.0108	0.3012	0.4996	0.020*	
O1B	0.45905 (14)	0.56597 (12)	0.19844 (12)	0.0189 (2)	
O2B	0.61849 (14)	0.75868 (13)	0.02759 (12)	0.0204 (2)	
H2OB	0.5906	0.6539	−0.0380	0.031*	
C1B	0.54652 (19)	0.72852 (18)	0.17064 (16)	0.0159 (3)	
C2B	0.5885 (2)	0.91565 (18)	0.29479 (16)	0.0173 (3)	
H2BA	0.5457	1.0160	0.2236	0.021*	
H2BB	0.7389	0.9603	0.3491	0.021*	
C3B	0.47841 (19)	0.89970 (17)	0.44459 (16)	0.0161 (3)	
H3BA	0.5266	0.8106	0.5238	0.019*	
H3BB	0.3356	0.8519	0.3947	0.019*	

### Atomic displacement parameters (Å^2^)

**Table:**

	*U*^11^	*U*^22^	*U*^33^	*U*^12^	*U*^13^	*U*^23^
O1A	0.0225 (5)	0.0180 (4)	0.0144 (4)	0.0084 (4)	0.0033 (3)	−0.0012 (3)
O2A	0.0287 (5)	0.0185 (4)	0.0129 (4)	0.0113 (4)	0.0033 (4)	−0.0023 (3)
C1A	0.0165 (6)	0.0157 (6)	0.0135 (6)	0.0019 (4)	0.0049 (4)	−0.0014 (4)
C2A	0.0218 (6)	0.0156 (6)	0.0151 (6)	0.0066 (5)	0.0044 (5)	−0.0013 (5)
C3A	0.0192 (6)	0.0155 (6)	0.0149 (6)	0.0050 (5)	0.0039 (5)	−0.0021 (5)
O1B	0.0248 (5)	0.0160 (4)	0.0166 (4)	0.0050 (4)	0.0078 (4)	−0.0008 (3)
O2B	0.0278 (5)	0.0164 (4)	0.0173 (5)	0.0022 (4)	0.0111 (4)	−0.0034 (3)
C1B	0.0157 (6)	0.0177 (6)	0.0140 (6)	0.0056 (5)	0.0028 (4)	−0.0009 (5)
C2B	0.0204 (6)	0.0149 (6)	0.0156 (6)	0.0029 (5)	0.0055 (5)	−0.0028 (5)
C3B	0.0185 (6)	0.0150 (6)	0.0138 (6)	0.0034 (5)	0.0039 (5)	−0.0019 (5)

### Geometric parameters (Å, °)

**Table:**

O1A—C1A	1.2199 (15)	O1B—C1B	1.2207 (15)
O2A—C1A	1.3237 (14)	O2B—C1B	1.3238 (15)
O2A—H2OA	0.8200	O2B—H2OB	0.8200
C1A—C2A	1.5007 (16)	C1B—C2B	1.5001 (16)
C2A—C3A	1.5220 (16)	C2B—C3B	1.5201 (17)
C2A—H2AA	0.9700	C2B—H2BA	0.9700
C2A—H2AB	0.9700	C2B—H2BB	0.9700
C3A—C3A^i^	1.528 (2)	C3B—C3B^ii^	1.525 (2)
C3A—H3AA	0.9222	C3B—H3BA	0.9537
C3A—H3AB	0.9462	C3B—H3BB	0.9224
			
C1A—O2A—H2OA	109.5	C1B—O2B—H2OB	109.5
O1A—C1A—O2A	123.52 (11)	O1B—C1B—O2B	123.41 (11)
O1A—C1A—C2A	124.18 (11)	O1B—C1B—C2B	124.33 (11)
O2A—C1A—C2A	112.29 (10)	O2B—C1B—C2B	112.25 (10)
C1A—C2A—C3A	114.73 (10)	C1B—C2B—C3B	115.27 (10)
C1A—C2A—H2AA	108.6	C1B—C2B—H2BA	108.5
C3A—C2A—H2AA	108.6	C3B—C2B—H2BA	108.5
C1A—C2A—H2AB	108.6	C1B—C2B—H2BB	108.5
C3A—C2A—H2AB	108.6	C3B—C2B—H2BB	108.5
H2AA—C2A—H2AB	107.6	H2BA—C2B—H2BB	107.5
C2A—C3A—C3A^i^	111.19 (13)	C2B—C3B—C3B^ii^	110.83 (12)
C2A—C3A—H3AA	110.2	C2B—C3B—H3BA	110.6
C3A^i^—C3A—H3AA	111.6	C3B^ii^—C3B—H3BA	108.1
C2A—C3A—H3AB	109.9	C2B—C3B—H3BB	109.3
C3A^i^—C3A—H3AB	106.7	C3B^ii^—C3B—H3BB	109.7
H3AA—C3A—H3AB	107.0	H3BA—C3B—H3BB	108.3
			
O1A—C1A—C2A—C3A	0.07 (17)	O1B—C1B—C2B—C3B	−11.15 (18)
O2A—C1A—C2A—C3A	179.19 (10)	O2B—C1B—C2B—C3B	169.89 (10)
C1A—C2A—C3A—C3A^i^	−172.28 (12)	C1B—C2B—C3B—C3B^ii^	−176.67 (13)

Symmetry codes: (i) −*x*, −*y*+1, −*z*+1; (ii) −*x*+1, −*y*+2, −*z*+1.

### Hydrogen-bond geometry (Å, °)

**Table:**

*D*—H···*A*	*D*—H	H···*A*	*D*···*A*	*D*—H···*A*
O2A—H2OA···O1A^iii^	0.82	1.82	2.6397 (13)	172
O2B—H2OB···O1B^iv^	0.82	1.82	2.6421 (13)	174
C2A—H2AB···O2A^v^	0.97	2.58	3.5415 (16)	171

Symmetry codes: (iii) −*x*, −*y*, −*z*; (iv) −*x*+1, −*y*+1, −*z*; (v) −*x*, −*y*+1, −*z*.
